# Initial Validation of the QUALity of Interactions Inventory (QUALII): Findings from the Expert Panel Survey

**DOI:** 10.1002/alz70858_097944

**Published:** 2025-12-24

**Authors:** Anju Paudel, Marie Boltz, Elizabeth Galik, Laura Elizabeth Pirkle Howd

**Affiliations:** ^1^ Pennsylvania State University, University Park, PA, USA; ^2^ University of Maryland Baltimore, Baltimore, MD, USA

## Abstract

**Background:**

Positive care interactions between staff and older adults in residential care settings such as Assisted Living (AL) can lead to positive outcomes such as fewer behavioral symptoms of distress and better quality of life among the residents living with dementia. There is a lack of comprehensive measure that can be used to both guide the desired practice of positive care interactions and evaluate the impact of practice, particularly in AL settings. QUALity of Interactions Inventory (QUALII)— a five domain 21‐item tool was developed as a comprehensive measure to support positive care interactions in dementia care in ALs. This study will describe the content validity of QUALII based on the Delphi survey with expert panel.

**Method:**

A Delphi survey approach was used with a panel of 10 clinical and academic experts in dementia care and communication; surveys were administered electronically. The quantitative ratings were used to compute Content Validity Index (CVI) for each item (I‐CVI) and overall scale (S‐CVI). The qualitative feedback and responses were reviewed using content analysis to inform item revisions.

**Result:**

The expert panel had diverse background (nursing, recreation/activity, dementia research, and clinical or program administration) and experience (four to 45 years) in gerontology and geriatrics. Following three rounds of the Delphi survey, the CVI values were: I‐CVI = 0.90 to 1.00 for item relevance, I‐CVI = 0.80 to 1.00 for item clarity, and S‐CVI = 0.93 for overall scale. Items related to supporting resident engagement and expression, individualizing communication approach, and managing pace of care during interactions were revised based on qualitative feedback from the experts.

**Conclusion:**

QUALII demonstrated excellent content validity following revisions. Upon successful field‐testing in AL settings, QUALII is expected to emerge as a comprehensive and psychometrically sound instrument that can be used to promote positive care interactions in dementia care in clinical settings such as AL.

